# PARRoT- a homology-based strategy to quantify and compare RNA-sequencing from non-model organisms

**DOI:** 10.1186/s12859-016-1366-1

**Published:** 2016-12-22

**Authors:** Ruei-Chi Gan, Ting-Wen Chen, Timothy H. Wu, Po-Jung Huang, Chi-Ching Lee, Yuan-Ming Yeh, Cheng-Hsun Chiu, Hsien-Da Huang, Petrus Tang

**Affiliations:** 1 Department of Biological Science and Technology, National Chiao Tung University, Hsin-Chu, 300 Taiwan; 2 Bioinformatics Center, Molecular Medicine Research Center, Chang Gung University, Taoyuan, Taiwan; 3 Institute of Biomedical Informatics, National Yang-Ming University, Taipei City, Taiwan; 4 Molecular Infectious Diseases Research Center, Chang Gung Memorial Hospital, Taoyuan, Taiwan; 5 Institute of Bioinformatics and Systems Biology, National Chiao Tung University, Hsin-Chu, 300 Taiwan; 6 Molecular Regulation & Bioinformatics Laboratory, Chang Gung University, Taoyuan, Taiwan

**Keywords:** Comparative transcriptome, Transcriptome quantification, De novo transcriptome assembly, Non-model transcriptome, Web service

## Abstract

**Background:**

Next-generation sequencing promises the *de novo* genomic and transcriptomic analysis of samples of interests. However, there are only a few organisms having reference genomic sequences and even fewer having well-defined or curated annotations. For transcriptome studies focusing on organisms lacking proper reference genomes, the common strategy is *de novo* assembly followed by functional annotation. However, things become even more complicated when multiple transcriptomes are compared.

**Results:**

Here, we propose a new analysis strategy and quantification methods for quantifying expression level which not only generate a virtual reference from sequencing data, but also provide comparisons between transcriptomes. First, all reads from the transcriptome datasets are pooled together for *de novo* assembly. The assembled contigs are searched against NCBI NR databases to find potential homolog sequences. Based on the searched result, a set of virtual transcripts are generated and served as a reference transcriptome. By using the same reference, normalized quantification values including RC (read counts), *e*RPKM (estimated RPKM) and *e*TPM (estimated TPM) can be obtained that are comparable across transcriptome datasets. In order to demonstrate the feasibility of our strategy, we implement it in the web service PARRoT. PARRoT stands for Pipeline for Analyzing RNA Reads of Transcriptomes. It analyzes gene expression profiles for two transcriptome sequencing datasets. For better understanding of the biological meaning from the comparison among transcriptomes, PARRoT further provides linkage between these virtual transcripts and their potential function through showing best hits in SwissProt, NR database, assigning GO terms. Our demo datasets showed that PARRoT can analyze two paired-end transcriptomic datasets of approximately 100 million reads within just three hours.

**Conclusions:**

In this study, we proposed and implemented a strategy to analyze transcriptomes from non-reference organisms which offers the opportunity to quantify and compare transcriptome profiles through a homolog based virtual transcriptome reference. By using the homolog based reference, our strategy effectively avoids the problems that may cause from inconsistencies among transcriptomes. This strategy will shed lights on the field of comparative genomics for non-model organism. We have implemented PARRoT as a web service which is freely available at http://parrot.cgu.edu.tw.

## Background

RNA-Seq has become a revolutionary tool for transcriptomic analysis with the coming-of-age high-throughput sequencing technologies [1]. Traditional Sanger sequencing of cDNA and EST libraries had been replaced by the RNA-Seq approach, which directly determines the cDNA sequence in a low-cost, high-throughput and quantitative manner. RNA-Seq has been applied in transcriptome studies aiming to provide a more precise and comprehensive measurement of the differential expression levels of transcripts, thus altering and broadening our insights on the complexity and extent of transcriptomics. For the organisms with reference genomes, a typical RNA-Seq data analysis procedure starts by mapping the short reads to the genomic or the annotated mRNA sequences [2–4]. A number of software packages have been developed for this purpose, including SOAPaligner [5], TopHat/Cufflinks [6, 7], Scripture [8], and ERANGE [2]. The mapping results between reads and transcripts can then be used to quantify the transcriptome and reveal the expression profiles. By comparing transcript profiles of organism, differences in molecular constituents of cells from different tissues, developmental stage, physiological conditions or treatments etc. can be revealed.

While the standard reference-based RNA-Seq analysis consists with mapping, quantifying transcripts and finding difference, the required reference sequences are rarely available for most species other than a handful of model organisms. To enable transcriptome studies in these organisms lacking proper reference genomes, several tools have been developed for *de novo* short read assembly such as Trans-ABySS [9], Velvet [10], Oases [11], SOAPdenovo-Trans [12], and Trinity [13] etc. Most of these tools were developed based on the De Bruijn graph which is the same algorithm used in *de novo* genome assembly but with slightly modified models for removing sequencing errors or dealing multiple linkages in the De Bruijn graph. Once assembled transcripts were generated, it become feasible to generate an expression profile from mapping reads to this assembled transcriptome. Several methods are proposed to estimate the expression level, such as RPKM (Reads Per Kilobase of transcript per Million mapped reads) [2], FPKM (Fragments Per Kilobase of transcript per Million mapped reads) [7] or TPM (Transcripts Per Million) [14] etc. which can then be used for identifying differentially expressed transcripts. Nevertheless, these methods can not directly apply to transcripts without known lengths. Therefore, the number of raw reads was used to represent the expression level. For example, Nagayasu E. et al. have utilized this *de novo* transcriptome assembly strategy and read counts to investigate transcriptomic changes along the four different developmental stages of *Strongyloides venezuelensis* [15]. Siebert S. et al. also performed similar analysis strategy to discover differentially expressed genes between feeding polyp and swimming medusa in siphonophore *Nanomia bijuga* [16].

In addition to a proper reference and method for quantifying expression level, another important issue of transcriptome analysis in non-model organism is the functional classification of these assembled transcripts. Several methods have been developed for annotating these contigs. Blast2GO [17, 18] established a standard functional annotation approach that has been adopted in gene function assignment of both model and non-model organisms since 2005. BLAST [19] search is the core of Blast2GO, which was the most time-consuming step of the annotation process. Though this drawback has been improved through grid computing, 24 h are still required with sufficient computing resources to obtain the BLAST results of 20,000 contigs, as suggested by previous study [20]. As BLAST has become a bottleneck for functional assignment of transcripts derived from high-throughput sequencing dataset, RAPSearch [21] uses a collision-free hash table to index the database sequences for searching and achieves a 20–90-fold speedup relative to BLAST. However, RAPSearch takes short reads generated from high-throughput sequencing datasets as its input material, thus limiting its maximum input length to approximately 300 bp, and is therefore not suitable for replacing BLAST because *de novo* assemblers usually generate contigs with N50 size longer than 500 bp. LAST [22] replaced the seed-and-extend approach of BLAST by an adaptive seed method and gave an approximately 50-fold increase in search speed. Despite computationally intensive steps is theoretically not a limitation anymore, implementing these solutions still requires certain computational knowledge and skills. Some web services such as rQuant.web [23], wapRNA [24] and FX [25] etc. are developed to provide user-friendly interface for RNA sequencing analysis. However most of these tools are designed for model organisms with reference genomes. Previously, we released FastAnnotator which is developed for non-model organism transcriptome annotation, but it didn’t include *de novo* assembly and transcript quantification [26].

Here we present an analysis strategy which generates virtual reference based on homologous sequences and includes novel methods for transcriptome quantification. We implemented our strategy in an automated Pipeline for Analyzing RNA Reads of Transcriptomes, namely PARRoT, which can provide annotated transcripts and corresponding expression profiles from two RNA-Seq datasets. PARRoT generates contigs assembled from two RNA-Seq datasets to obtain a comprehensive and integrated reference transcriptome. Homologous sequences and functional annotation of these assembled transcripts are searched in the NCBI-nr [27] and SwissProt [28] databases by the modified robust homology search package LAST. PARRoT reconstruct virtual transcripts based on search results. To further access and quantify transcripts from different datasets, PARRoT maps RNA-Seq reads from each dataset back to the virtual transcripts, which makes it possible to derive read counts, normalized estimated TPM and RPKM values. These expression values can be downloaded for further differential gene expression analysis. PARRoT also offers plots which reveal the enriched/suppressed Gene Ontology (GO) [29] terms between the two transcriptome datasets. An user-friendly interface is available to classify, filter and compare the annotated contigs, virtual transcripts according to gene expression levels and GO terms. All these analyzed results are formatted as tab-delimited text files and compressed into a file for download. PARRoT is freely accessible through http://PARRoT.cgu.edu.tw.

## Methods

### Analysis strategy

**Fig. 1:**
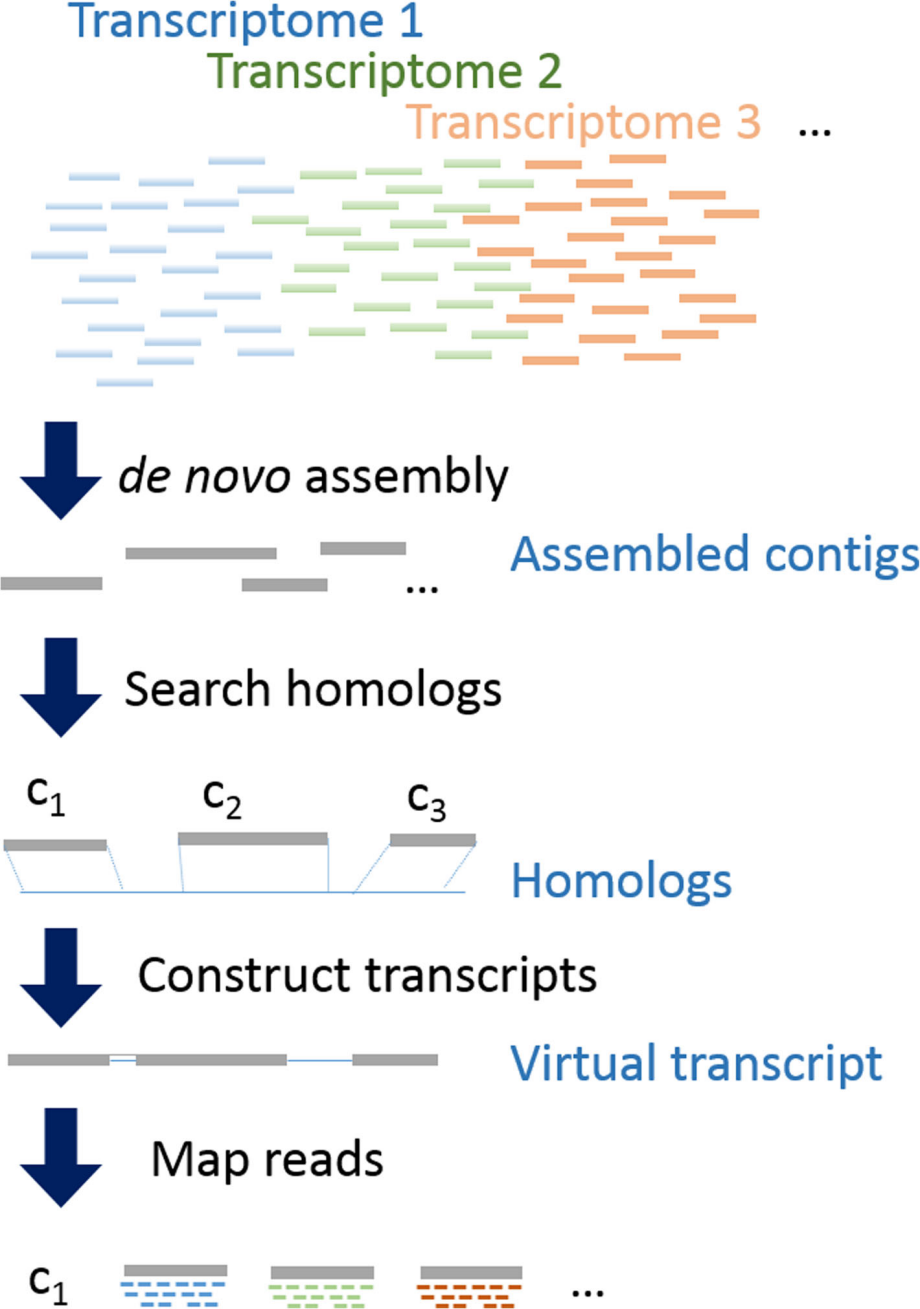
Analysis strategy for quantifying transcriptomes without a reference genome. All reads from different transcriptomes are pooled together for *de novo* assembly. Assembled contigs are used for searching homologs. Contigs which are matched to the same homolog are used to construct a virtual transcript for later use in quantification of expression. The sequencing reads are mapped to the virtual transcripts. Expression level for each virtual transcript are represented as mapped read count, estimated RPKM and estimated TPM based the mapping results

In order to quantify transcriptome from RNA-Seq data of non-model organisms, we propose an analysis strategy (Fig. 1) which can generate a virtual transcriptome reference and quantify expression profiles. Our strategy first performs *de novo* assembly for all reads from multiple RNA-Seq data and search homologs for these assembled contigs. Contigs belong to the same homolog are clustered together as a virtual transcript. After contigs were clustered according to the homolog search, reads from each RNA-Seq dataset are mapped to these virtual transcripts. After that, we propose three different expression quantification methods. The first one is read counts, shorten as RC for each contigs which is the number of reads mapped to that particular contig as used in previous studies [15, 16]. As shown in Equation 1, n_j_ represents the number of reads mapped to contig j. The second one is estimated RPKM (eRPKM) for each virtual transcripts, which is actually a unit similar to the normal RPKM, only that we used a virtual transcript instead of an annotated transcript. The formula for calculating eRPKM takes all contigs belonging to the same virtual transcript to estimate the expression level of that virtual transcript. Similar to RPKM, the denominator includes the number of all the mapped reads and a sum-up of lengths from assembled contigs belonging to that particular virtual transcript. Given a total of m virtual transcripts, for each transcript x, eRPKM is derived from Equation 2 in which k_x_ representing the number of contigs belonging to the virtual transcript x, n_x,i_ representing the number of reads mapped to mapped to the i^th^ contig belonging to the transcript x, l_x,i_ representing the length of the i^th^ contig belonging to transcript x. We also propose estimated TPM (eTPM) for each virtual transcript. The eTPM is calculated as Equation 3 in which the numerator is the number of transcript count for that particular virtual transcript and denominator is the total virtual transcript count.

1 $$ {\mathrm{RC}}_j = \kern0.5em {n}_j $$

2 $$ {\mathrm{eRPKM}}_{\mathrm{x}}=\frac{10^9\ast {\sum}_{i=1}^{k_x}{n}_{x,i}}{\ {\sum}_{i=1}^{k_x}{l}_{x,i}\ast {\sum}_{t=1}^m{\sum}_{i=1}^{k_t}{n}_{t,i}\ } $$

3 $$ {\mathrm{eTPM}}_{\mathrm{x}} = \frac{10^6 \ast \frac{\ {\sum}_{i=1}^{k_x}{n}_{x,i}}{\sum_{i=1}^{k_x}{l}_{x,i}\kern0.5em }}{\sum_{t=1}^m\ \frac{\ {\sum}_{i=1}^{k_t}{n}_{t,i}\ }{\sum_{i=1}^{k_t}{l}_{t,i}\ }\ } $$

### PARRoT website

**Fig. 2:**
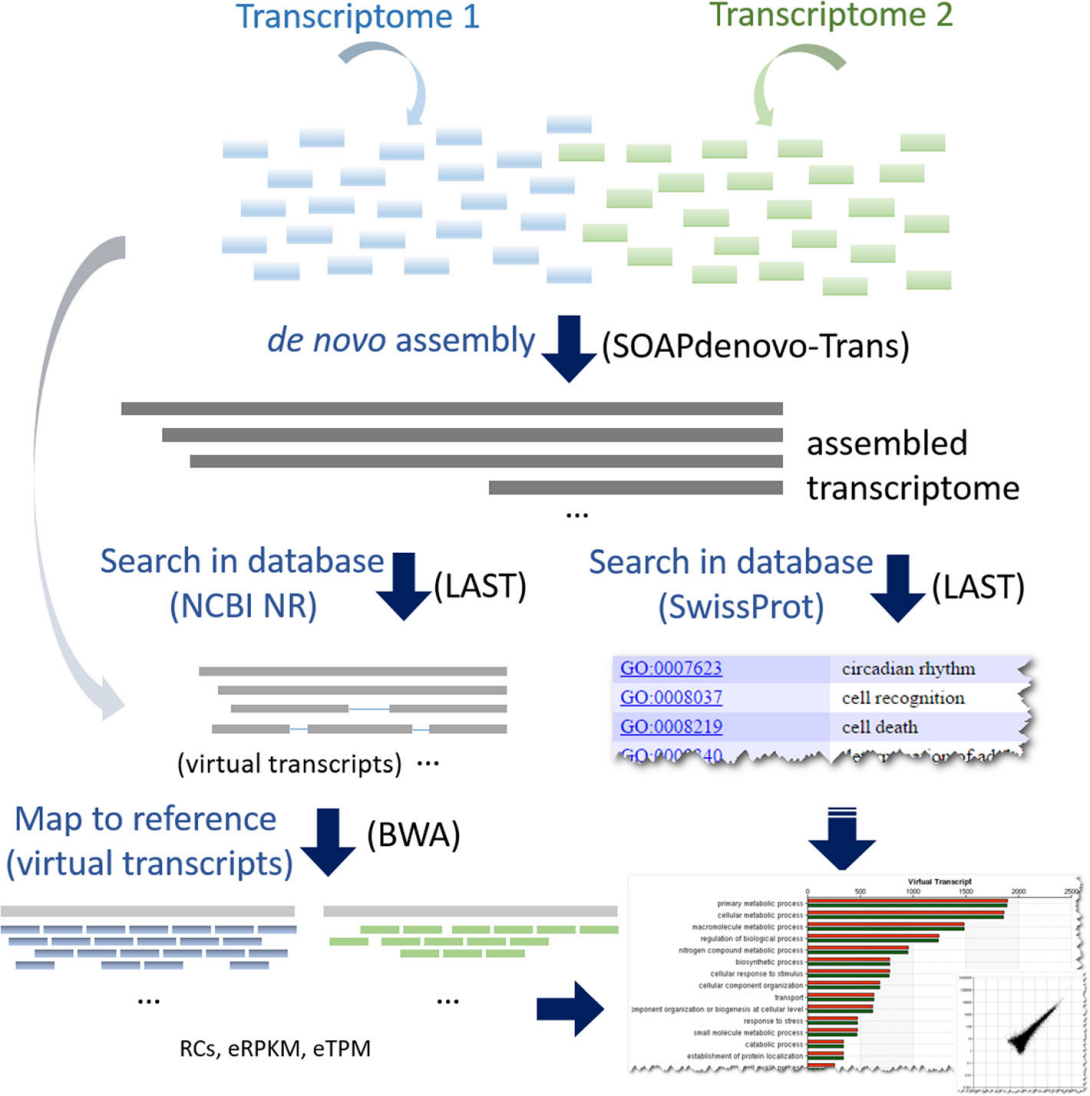
PARRoT workflow. PARRoT includes the following analysis steps: 1) *de novo* assembly of pooled RNA-Seq data; 2) functional annotation and homolog search; 3) generated virtual transcripts; 4) mapping the sequence reads; 5) quantification of each transcript contig by calculating the RC (number of mapped read counts), eRPKM (estimated RPKM) and eTPM (estimated TPM) in each transcriptome dataset; and 6) show the expression level of contig or virtual transcript for two datasets together with their functional annotations

We implemented our strategy in a web-service, Pipeline for Analyzing RNA Reads of Transcriptomes (PARRoT). The analysis includes the following six steps. Step 1, PARRoT utilizes SOAPdenovo-Trans [12] for *de novo* assembly and constructs assembled contigs for a pooled dataset of combination from two RNA-Seq datasets uploaded with the default parameter. Step2, in order to provide a better biological explanation for the differentially expressed genes, PARRoT annotates the reference contigs by homology search against SwissProt [28] and associates GO terms with contig and virtual transcripts. PARRoT also searches these assembled contigs against NCBI non-redundant protein database [27] to find homologs for each contig with LAST [30]. Step 3, PARRoT generates virtual transcripts based on homolog search results from step 2. Step 4, after contigs are clustered as virtual transcripts, PARRoT maps RNA-Seq reads to these virtual transcripts with BWA [31]. Step 5, PARRoT calculates RC, eRPKM and eTPM based on equation 1, 2 and 3 described previously for each virtual transcript in each dataset based on their mapping results respectively. Step 6, all quantification results are presented in an interactive user interface in which user can select different annotation levels. All GO-terms were downloaded from GO official website (http://www.geneontology.org) and pre-computed to reconstruct the hierarchical relationships. Contigs matching the same GO are then clustered for calculating total contig counts and the associated transcript expression level. For a better understanding of GO annotations, various levels of the GO terms and three quantification results are provided altogether. By selecting different level of GO annotations, user can explore the difference and similarity between two uploaded datasets. All the annotation, *de novo* assembled contigs and quantification results can be downloaded as a zip file. The complete analysis workflow of PARRoT is illustrated in Fig. 2.

### Web server information

PARROT runs on a Linux Ubuntu 64-bit server, which houses a 4-core Intel® Core-i7® Processors (4820 K, 3.7GHz) and 64GB RAM and is installed in the Chang Gung Bioinformatics Center. Data processing is performed using Perl, Python and Linux shell scripts. The web interface is generated using PHP and ChartDirector for PHP. Condor is used as our workload management system for compute- and memory-intensive jobs (http://www.cs.wisc.edu/condor). Based on the estimation of two paired-end datasets of ~100 million reads, PARROT can finish the whole analysis in 3 h by using 2 CPU cores.

## Results

In order to solve the problem of lacking proper reference for non-model organism transcriptome analysis, we propose an analysis strategy including pooled-assembly, clustering contigs on virtual transcripts and several quantification methods. We implement this approach as a web-service, PARRoT. Users who want to apply our analysis strategy can upload their transcriptome sequencing data to PARRoT. Currently, PARRoT accepts two transcriptomes, either sequenced by single-end or paired-end by NGS. PARRoT accepts two RNA-Seq datasets in various compressed formats (zip or gzip) containing the FASTQ file as input. Once the input is successfully uploaded, PARROT will start the analysis process and return a page containing a unique job identifier that can be used later to retrieve the results. PARRoT assembles, quantifies and annotates contigs with default parameters in SOAPdenovo-Trans, BWA and several in-house scripts. Default parameters in BWA and SOAPdenovo-Trans were used except the k-mer value used in SOAPdenovo-Trans. In order to detect the transcripts in low expression levels, PARRoT uses a small k-mer value, as 31, as suggested in the SOAPdenovo-Trans tutorial. PARRoT is designed for comparing the transcriptomes in two different conditions from the same species and is not recommended for comparative genomics between species. It is worth mentioning that PARRoT takes all uploaded sequenced into *de novo* assembly. Hence users can/should apply their own reads selection criteria such as trimming and removing low-quality reads before they upload reads.

After reads are assembled into contigs, these contigs are used to search against the NCBI non redundant database which may include homologs for these contigs. Contigs came from the same transcript are likely to have the same best hit due to the sequence similarity between homologs. These contigs hit the same transcripts are then used to construct virtual transcript which will then be used for quantifying expression levels as described in implementation section. In addition to quantify expression level for transcriptome. We also include the functional annotation in PARRoT. Once the contigs are assembled, PARRoT will search these contigs against SwissProt database and using their hits to provide clues for potential function of these contigs. As a web-service for quantifying expression level and annotating expressed contigs, the output of PARRoT includes summary of assembly result, number of contigs overlapped or unique in two transcriptomes, summary of contig expression level in RPKM, summary of gene annotation, functional annotation for contigs and plots summarizing the expression level of each functional category. All these annotation, RC, eRPKM, eTPM values can be downloaded as txt file for further analysis.

**Fig. 3:**
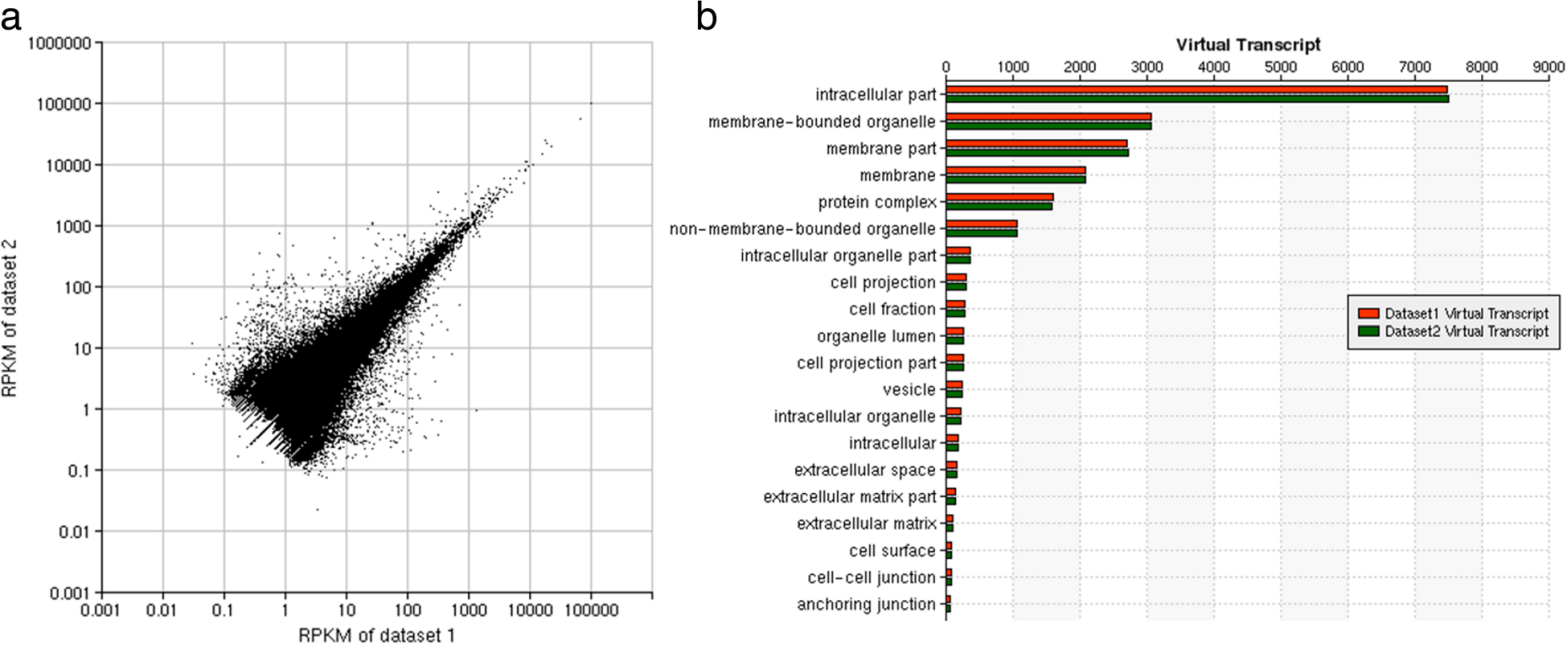
Expression level and number of virtual transcriptomes from Cnidaria. **a** For each contigs generated from pooled assembly, the expression level in RPKM is calculated for each virtual transcript. This plot shows that the expression level for most contigs from these two transcriptome datasets are similar to each other. **b** Number of virtual transcripts in each GO cellular component category in each transcriptome. After searching the most similar sequence of contigs in NCBI NR and Swiss-Prot databases, the GO annotations for the best hits are used to provide functional annotations for the assembled contigs. For all contigs hitting the same transcript in NR database, PARRoT quantify the expressions from them altogether because they are likely to come from the same transcript which is called as a virtual transcript in the plot. PARRoT calculates how many virtual transcripts were found for each GO category in each transcriptome

**Fig. 4:**
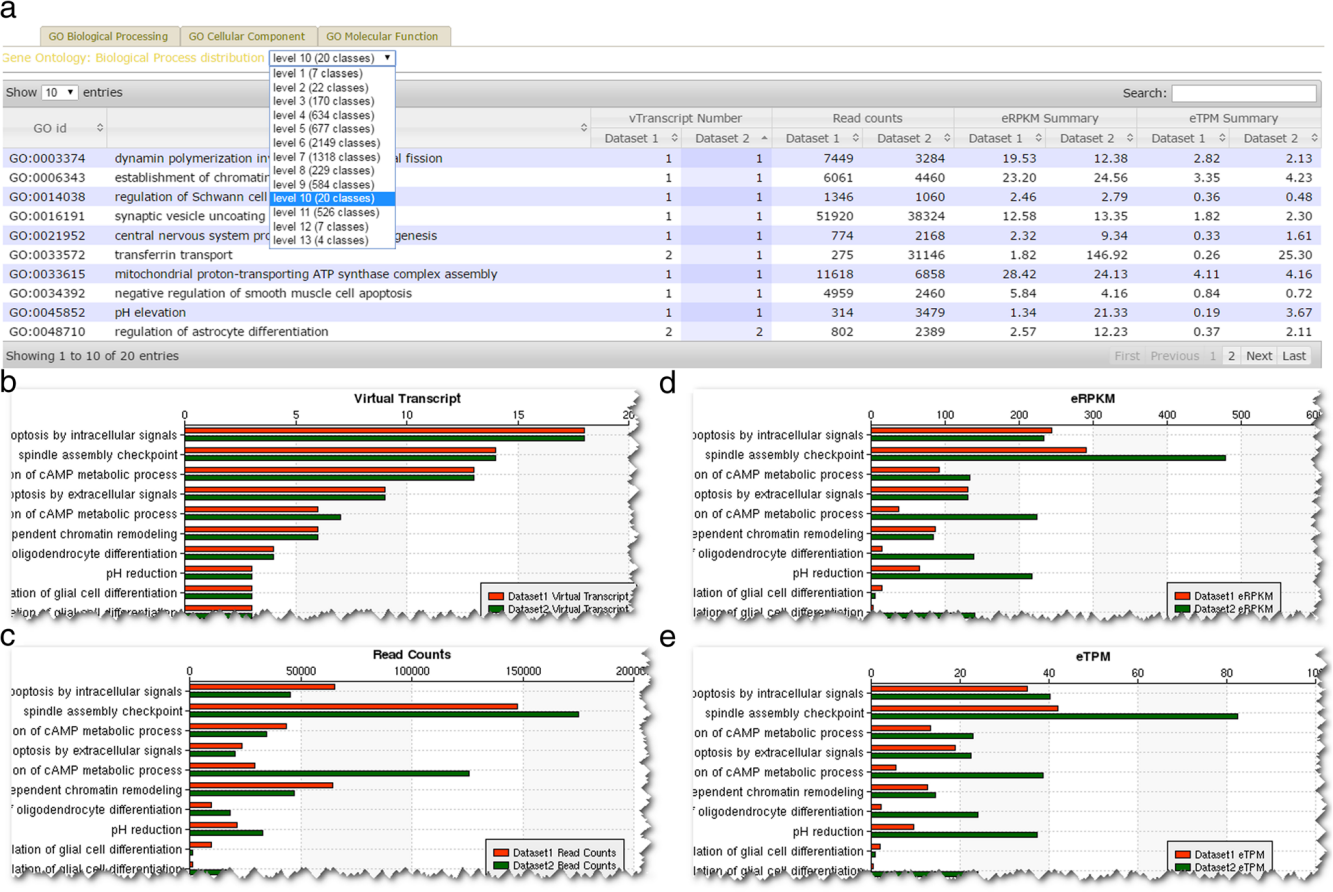
Cumulative expression level for GO terms. **a** Users can select different level for GO terms. PARRoT includes pre-computed tree structure for GO terms and users can selection the level from the dropdown list. Once the level is changed, both the plot and table will change with new data corresponding to whatever the level users select. **b** Number of virtual transcripts belong to the GO terms. **c** Number of raw counts mapped to the virtual transcripts belonging to the GO terms. **d** Sum up of all estimated RPKM (eRPKM) for virtual transcripts belonging to the GO terms. **e** Sum up of all estimated TPM (eTPM) for virtual transcripts belonging to the GO terms

To exhibit the performance and output of PARRoT, we used RNA-Seq data for transcriptome in the Siphonophore *Nanomia bijuga* as a demostration dataset [16]. Two files containing 33,130,955 and 33,291,056 single-end RNA sequencing reads (SRR081276 and SRR089297) were downloaded from the NCBI GEO database. It took PARRoT 1 h 26 mins to finish the assembly, annotation and quantification. There are a total of 492,130 contigs (range: 32 bp ~ 20,960 bp) assembled from these two transcriptomes and the distribution of RPKM shows that the majority of these contigs have similar expression levels (Fig. 3a). In addition to quantification for expression profiles, PARRoT also provides a high level of the flexibility in analyzing the GO results. Most of these transcripts we found are located in the intracellular part, followed by membrane-bounded organelle, membrane part and protein complex, etc. (Fig. 3b). Users can navigate the GO information at a specific level of interest and customize their analysis depending on the project aims. Few other existing software or pipelines offer such the multi-layer comparative functionality. Because of the unique flexibility in navigating different hierarchies of the GO layers, PARRoT allows users to further explore the functional groups in various GO layers with a cumulative set of differentially expressed genes (Fig. 4a). This provides an in-depth and comprehensive insight on the potential activation or inactivation of biological functions. Detail information for this demo dataset can be found in demo example 2 on the PARRoT Demo page.

PARRoT also supports paired-end reads. Hence we further included demo dataset 3 and 4 which were transcriptome from fat body tissues in the mountain pine beetle [32]. Demo dataset 3 includes two transcriptomes sequenced by Illumina GA and having 5,580,576 and 9,751,797 paired-end reads from the fat body and midgut, respectively. Transcriptomes included in demo dataset 4 are from the same sample as dataset 3 and sequenced with Illumina HiSeq 2000 and having 76,780,490 and 52,369,061 paired-end reads, respectively. PARRoT took 30 mins and 2 h 55 mins to finish the analysis of demo dataset 3 and demo dataset 4. We examed the biological process distribution to understand how RC, eRPKM and eTPM work in demo dataset 4. Even though the number of virtual transcripts found in the two datasets were similar, the RC for fat body was almost always larger than midgut (Fig. 4a and b). This does not really result from difference in expression between these tissues, but rather the difference in the number of reads in original input. However, the overwhelming higher expression patterns was somewhat adjusted when using eRPKM or eTPM as unit to measure expression level (Fig. 4d and e). It’s worth mentioning that the result shown on PARRoT is a sum up from many virtual transcripts and the exact values of eRPKM and eTPM for most virtual transcripts are even closer. As several examples shown in Fig. 4a, for most rows having vTranscript number equal to 1 in both datasets actually have similar eRPKM or eTPM values comparing to RC values. The results for these two demo dataset are listed on PARRoT demo page.

Users can get familiar with the output and analysis steps of PARROT by using two 90 bp paired-end RNA-Seq datasets from the parasitic protozoan *Trichomonas vaginalis*. In this dataset, 48,196 gene contigs were generated after de novo assembly for the subsequent analyses with an N50 of 562. There are 88.71% assembled contigs successfully mapped to the SwissProt or the NCBI-nr databases. All the pre-computed demonstrative transcriptomic comparison results allow users to navigate the analytical outputs on the PARROT website, before uploading their datasets of interests.

## Discussion

We proposed to assemble RNA-Seq reads from different transcriptomes together to reconstruct a comprehensive transcriptomic contigs. These contigs can then be used to find virtual transcripts from known transcript database. In addition to putative functional annotation, these virtual transcripts also provide the possibility to quantify the expression level of each transcriptome by the same reference. Conventionally, the different transcriptomic datasets are *de novo* assembled independently and results in several unique transcriptomic datasets. However, this independent assembly strategy may result in less complete gene contigs or partial contig segments due to insufficient sequencing coverage. Meanwhile, it is not feasible to generate comparable expression levels by applying same quantification method to each different reference datasets. Therefore, even though the pooled assembly strategy may result in some wrong assembly contigs especially for these genes having many alternative splicing form. Still this kind of pooled assembly make quantifying expression level using the same transcript reference possible and make us using it for analyzing transcriptomes for species without reference genome information. In addition to pooled assembly, we further propose several modified quantitative methods for quantifying the expression level using the idea of virtual transcripts. To our knowledge, these quantification methods provide comparable quantification standard to compare expression levels between different transcriptome datasets for the first time.

We implement our analysis strategy by constructing a web-server, PARRoT, in which we demonstrate that expression level in RC, eRPKM and eTPM can be useful for comparing between two transcriptomes. PARRoT is also the first web-service designed for comparing expression profiles between transcriptomes without requiring the prior knowledge for reference genome. Although there are web applications available for analyzing RNA-Seq data [24, 33], few of them were designed for analyzing *de novo* transcriptome for non-model organisms [26]. Meanwhile, most of the available *de novo* assemblers were designed for local execution only, lacking downstream analyses such as aligning the RNA-Seq reads back to *de novo* assembled transcripts, analyzing differentially expressed transcripts between datasets and annotating the *de novo* assembled transcripts. Therefore, we believe PARRoT will be an unique and invaluable tool for non-model organism transcriptome studies.

We also apply our quantification method in GO terms. The conventional GO annotation analysis provides only qualitative information, without knowing the quantitative information of differential expression profiles between the two compared transcriptomes. In the functional comparison, PARRoT provides potential estimation for each GO terms in which the number of virtual transcripts belonging to the same GO annotation or RC, eRPKM and eTPM for different virtual transcripts having the same pathway or GO annotation are summed up. These estimations can provide some clues for estimating the overall expression of different functional categories in the two input datasets. Furthermore, the RC, eRPKM and eTPM for each virtual transcript in either input datasets are available for download. Considering that the activities and importance of genes within one pathway are surely different, users are encouraged to download detailed data for further investigation of the changes for each contig.

PARRoT annotates transcripts through homology search and assigns functional annotation from the most similar sequences as the annotation of the newly assembled transcript. During the annotation process, PARROT maps sequence contigs back to known proteins databases by employing LAST [22] instead of the most widely used alignment tool, BLASTx, for two major reasons. Firstly, LAST is more likely to find distant homologs. BLASTx searches sequences based on sequence similarity and is much more restrictive in finding evolutionarily distant relatively related sequences. In contrast, LAST searches similar sequences by adaptive seeds that take the rareness of sequence into consideration. This strategy is more likely to find evolutionarily conserved sequences and overcomes the challenge of lower sequence similarity between distantly related homologs. This type of homolog search for dissimilar sequences is extremely important especially for RNS-Seq data that come from a relatively unexplored organism or an environmental sample. Secondly, LAST is exponentially faster than BLASTx. We tested the performance of LAST and BLASTx on a Linux 64-bit server with 10,000 contigs (total 7,896,991 bases). BLASTx took 18 days 6 h 24 min to search against the NCBI nr database while LAST only took less than 10 min. Results from these two tools are comparable despite the speed difference, sharing 82% of the contigs have the same best-hits. LAST provides comparable sensitivity as BLASTx in the detection of similar sequences. Therefore, we employ LAST in PARROT.

The well know annotation tool, BLAST2GO is widely used to annotate sequences. However, BLAST2GO is no longer free now for all its annotation functions and is highly time consuming. We compared performance and results of our pipeline with BLAST2GO with an input of 39,914 contigs (total 24, 168,315 bases). It turns out that BLAST2GO took approximately 6 h while our pipeline took less than 3 min on a Linux 64-bit server. As for the results, PARROT annotated 46.26% of contigs while BLAST2GO annotated 64.43% of contigs. Even though our pipeline has a lower annotation rate, our searching strategy annotate contigs based on highly similar sequences. On the other hand, BLAST2GO includes heuristic methods to calculate confidence score for potential function for all sequences even for those having no obvious similar sequences in the database. That enable BLAST2GO to have a higher annotation rate with an estimated accuracy around 65–70% [18]. We chose to use our own pipeline to provide the most confident annotation results in a reasonable computing time.

In addition to the annotation strategy, PARRoT only annotates contigs longer than 200 bp in order to avoid incorrect annotation for partial transcripts. Those short contigs can at most encode proteins that are 66 amino acids in length, even if we assume there was no untranslated region. There is indeed a possibility that PARRoT loses some full transcripts which are really short and which encode short proteins. To assess this possibility, we investigate the length distribution of proteins. According to protein sequences downloaded from NCBI genomes for *Homo sapiens* and *C. elegans*, there are only 0.65 and 1.65% of proteins shorter than 67 amino acids, respectively. Even though it’s been know that the mean protein length of eukaryotes is actually 40–60% longer than that of prokaryotes, in previous study it’s estimated that there are only 3.62% of proteins shorter than 67 amino acids [34].

## Conclusions

Inspiring by the idea of RPKM and TPM used for RNA-Seq in genomes having reference sequences and annotations, we proposed a new analysis strategy together with quantification methods for compare transcriptomes from species without a proper reference genome. We also constructed a webserver PARRoT to elucidate our strategy. PARRoT is an ultrafast and useful tool that can process two RNA-Seq datasets from sequencing reads and provide a solutions for comparing their expression profiles. The web interfaces are intuitively designed to provide user-friendly navigation of results and the relevant downloadable results. Users don’t need *a priori* knowledge of data processing of high-throughput DNA sequencing or relevant bioinformatics skills. PARRoT takes only 0.5 h and 3 h to finish a *de novo* assembly and generate the subsequent annotations of two RNA-Seq paired demo datasets of around 10 million paired-end reads and 100 million paired-end reads respectively. PARROT is now the only web-based suite providing a complete solution for performing *de novo* assembly, functional assignment, and differential gene analysis of two RNA-Seq datasets.
